# Predicting Treatment Response of Neoadjuvant Chemoradiotherapy in Locally Advanced Rectal Cancer Using Amide Proton Transfer MRI Combined With Diffusion-Weighted Imaging

**DOI:** 10.3389/fonc.2021.698427

**Published:** 2021-07-01

**Authors:** Weicui Chen, Liting Mao, Ling Li, Qiurong Wei, Shaowei Hu, Yongsong Ye, Jieping Feng, Bo Liu, Xian Liu

**Affiliations:** ^1^ Department of Radiology, The Second Affiliated Hospital, Guangzhou University of Chinese Medicine, Guangzhou, China; ^2^ Department of Pathology, The Second Affiliated Hospital, Guangzhou University of Chinese Medicine, Guangzhou, China

**Keywords:** Amide proton transfer, diffusion-weighted imaging, neoadjuvant chemoradiotherapy, locally advanced rectal cancer (LARC), treatment response

## Abstract

**Objective:**

To evaluate amide proton weighted (APTw) MRI combined with diffusion-weighted imaging (DWI) in predicting neoadjuvant chemoradiotherapy (NCRT) response in patients with locally advanced rectal cancer (LARC).

**Methods:**

53 patients with LARC were enrolled in this retrospective study. MR examination including APTw MRI and DWI was performed before and after NCRT. APTw SI, ADC value, tumor size, CEA level before and after NCRT were assessed. The difference of the above parameters between before and after NCRT was calculated. The tumor regression grading (TRG) was assessed by American Joint Committee on Cancer’s Cancer Staging Manual AJCC 8th score. The Shapiro-Wilk test, paired t-test and Wilcoxon Signed Ranks test, two-sample t-test, Mann-Whitney U test and multivariate analysis were used for statistical analysis.

**Results:**

Of the 53 patients, 19 had good responses (TRG 0-1), 34 had poor responses (TRG 2-3). After NCRT, all the rectal tumors demonstrated decreased APT values, increased ADC values, reduced tumor volumes and CEA levels (all p < 0.001). Good responders demonstrated higher pre-APT values, higher Δ APT values, lower pre- ADC values and higher Δ tumor volumes than poor responders. Pre-APT combined with pre-ADC achieved the best diagnostic performance, with AUC of 0.895 (sensitivity of 85.29%, specificity of 89.47%, p < 0.001) in predicting good response to NCRT.

**Conclusion:**

The combination of APTw and DWI may serve as a noninvasive biomarker for evaluating and identifying response to NCRT in LARC patients.

## Highlights

- After NCRT, All the rectal tumors demonstrated decrease APT values, increased ADC values, reduced tumor volumes and CEA levels.
- Good responders to NCRT demonstrated higher pre-APT values, higher Δ APT values, lower pre-ADC values and higher Δ tumor volumes than poor responders.
- A combination of APT and ADC values before NCRT exhibited a good diagnostic performance in predicting a good response to NCRT (with AUC of 0.895, sensitivity of 85.29% and specificity of 89.47%).

## Introduction

Currently, preoperative neoadjuvant chemoradiotherapy (NCRT) followed by total mesorectal excision surgery is the standard treatment protocol for locally advanced rectal cancer (LARC) (1). NCRT aims to downstage the tumor, enable complete surgical resection, and reduce the risk of recurrence and metastases (2). Some strictly selected patients can even achieve complete clinical response with a “wait and see” policy after NCRT, avoiding surgical treatment (3). However, significant unexplained variation remains in the responses to NCRT. A series of clinical trials demonstrated that 8% to 27.5% of patients who achieved pathologic complete response (pCR) after NCRT have a better long-term outcome, lower recurrence risk, and improved overall survival (4). Approximately 54–75% of patients had tumor downstaging, and the remainder had no treatment response. Therefore, predicting the response to NCRT is important for patients with potentially curable LARC who wish to explore personalized treatment to improve their therapeutic outcomes.

MRI plays an important role in the therapeutic assessment of rectal carcinoma, particularly beneficial to surgical planning and optimize treatment strategies for patients with different responses (5). MR-based tumor regression grade (mrTRG) was validated to be associated with disease-free and survival outcomes by The MRI and Rectal Cancer European Equivalence (MERCURY) trial (6). However, conventional T2WI MRI is limited by its inability to differentiate post-therapeutic edema and fibrosis from residual tumor tissue. Morphological parameters were also proved to be helpful in assessing pCR. Some studies demonstrated a significant correlation between tumor volume reduction and pCR (7–9). Furthermore, Fiorino C et al. introduced an early regression index based on a logarithmic transformation of change in tumor volume (10). This new predictive index showed great discriminative power in evaluating tumor response to NCRT and long-term disease-free survival (10, 11). Functional MRI, such as diffusion-weighted imaging (DWI) and dynamic-contrast-enhanced MRI (DCE-MRI) can provide additional physiological information about a tumor’s cellular environment and perfusion characteristics, offering great potential to assess the therapeutic response of rectal cancer (12–15). DWI has been widely used in the evaluation of tumor response to NCRT in rectal cancer, as its capability of providing information on tumor cellular architecture. However, results regarding the use of ADC in predicting the NCRT response have been inconsistent. This variation may be due to a lack of standardized imaging, acquisition techniques, and analysis methods (5, 12).

Lately, considerable progress has been made in devising radiomics or deep learning techniques to assess the treatment response of NCRT in LARC (13, 16, 17). Horvat et al. found that radiomics provided a significantly greater diagnostic capability than T2WI or DWI alone when using a random forest classifier to investigate T2WI-based radiomics while evaluating complete clinical response in rectal cancer patients after NCRT (17). Zhang et al. used a deep learning model based on diffusion kurtosis MRI to predict pCR in assessing the response of LARC after NCRT. The deep learning model showed good diagnostic performance and aided radiologists in assessing pCR (18). However, these extracted radiomics features depend on image acquisition, reconstruction, and processing methods, which naturally vary among different institutes and operators (19). Their clinical application is restricted by reproducibility and reliability. Besides, the deep learning model is typically too complex to interpret the relationship between extracted properties and tumor biology (20). To our knowledge, a reliable classification system has yet to be developed.

Amide proton transfer-weighted (APTw) MRI is a molecular MRI technique based on chemical exchange saturation transfer (CEST), which is achieved through qualifying the exchange between amide proton (-NH) groups of endogenous mobile proteins/peptides and bulk water (21). The APTw signal is related mainly to the concentration of mobile proteins, making it beneficial for assessing tumor grade and differentiation (22–24). Clinical APTw imaging has also shown promise in tumor monitoring in gliomas. Several studies have reported that APTw imaging helps differentiate between pseudo-progression, radiation necrosis, and tumor recurrence in gliomas (25)—it is superior to conventional MRI contrasts as well as to advanced functional imaging methods such as perfusion (dynamic susceptibility contrast and dynamic-contrast-enhanced) and spectroscopy (24, 26–28). However, only one study assessed APTw MRI in evaluating the effect of chemoradiotherapy in LARC (29).

Therefore, considering the characteristics of APTw imaging and DWI, we aimed to investigate the value of APTw imaging combined with DWI for predicting the treatment response of NCRT in patients with LARC.

## Materials and Methods

### Participants

The current study was approved by the Medical Ethics Committee of our hospital (Ref. No. YE2019-274-01) and written informed consent was obtained from each patient before the MR examination. Between February 2019 to May 2021, 88 consecutive patients diagnosed with LARC were included. All the patients received preoperative NCRT followed by TME surgery.

**Figure 1:**
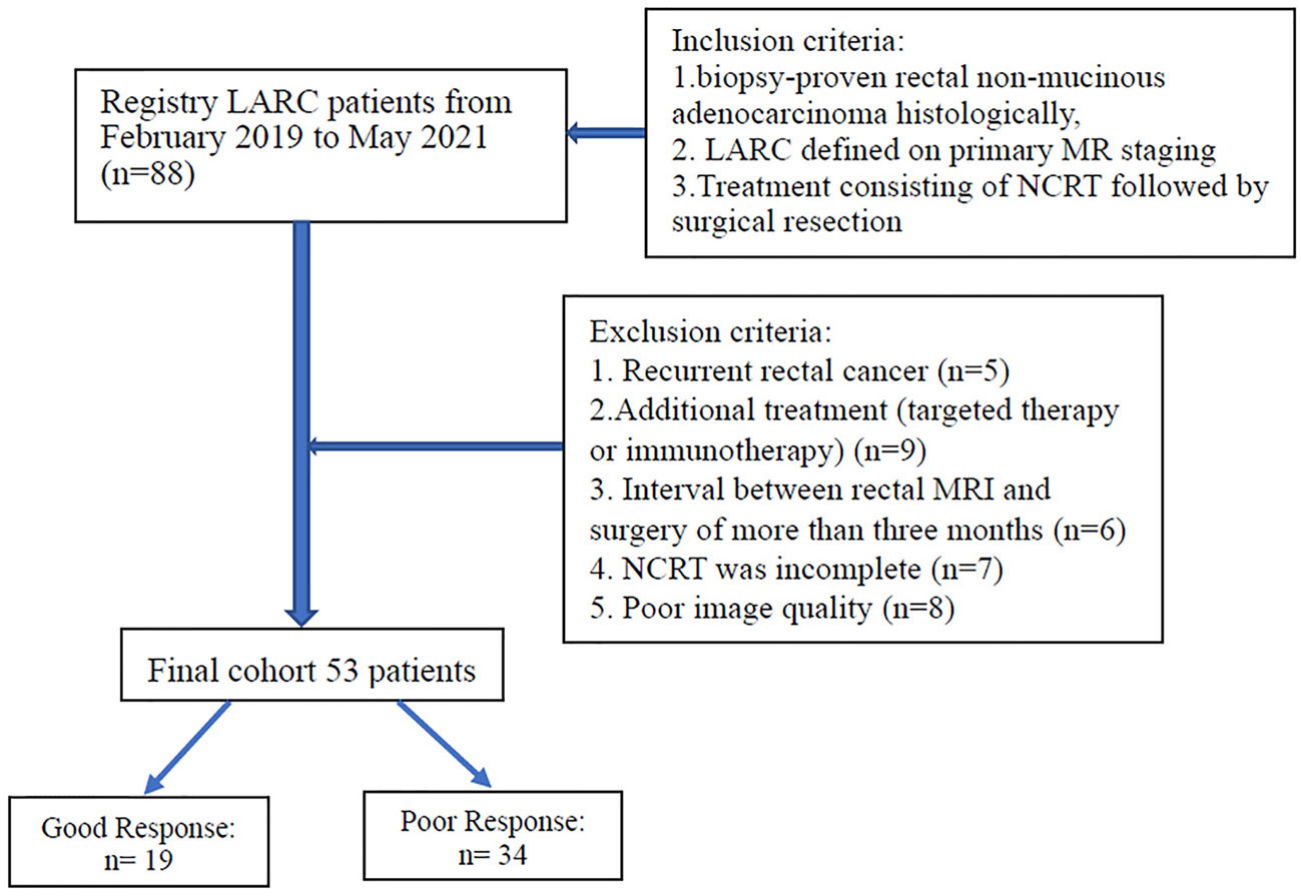
Flowchart of patient selection.

The inclusion criteria were as follows: 1) biopsy-proven rectal non-mucinous adenocarcinoma histologically, 2) LARC (category cT3 or cT4, node-positive status) defined on primary MR staging, 3) treatment consisting of NCRT followed by surgical resection. The exclusion criteria were as follows: 1) recurrent rectal cancer, (b) additional treatment (targeted therapy or immunotherapy), 3) interval between restaging rectal MRI and surgery of more than three months, 4) NCRT was incomplete, 5) poor image quality (included motion artifacts and image distortion from susceptibility effect due to bowel gas). **Figure 1** displays the patient selection flowchart.

### NCRT Treatment

NCRT consisted of 45–50 Gy of radiation delivered in daily doses of 1.5 or2 Gy, five fractions per week, and concomitant chemotherapy. Neoadjuvant chemotherapy regimens were as follows: (1) oxaliplatin 130 mg/m^2^ (iv gtt, d1) and capecitabine 1000 mg/m^2^ (per os, bid, d1-14) every three weeks for 6–8 courses (XELOX) in eighteen patients; (2) folinic acid 200 mg/m^2^ (d1), fluorouracil 400 mg/m^2^ (d1), fluorouracil 2400 mg/m^2^ (d1-d2), and oxaliplatin 85mg/m^2^ (d1) for 3–7 courses (mFOLFOX6) in ten patients; (3) capecitabine 1250 mg/m^2^ (per os, bid, d1-d14) for 3–6 courses in 25 patients. Surgery with total mesorectal excision was performed within 6–8 weeks after the completion of NCRT.

### MRI Protocol

All participants received two MRI examinations: the first within one week before NCRT (pre-NCRT MRI) and the second within one week before surgery (post-NCRT MRI).

All MRI examinations were performed on a 3.0T MRI scanner (Ingenia, Philips Healthcare, Best, the Netherlands) using a 32-channel phased-array coil. A glycerin enema was performed before the examination to reduce distortion due to gas in the rectum. At 30 min before the MR examination, 5 mg of raceanisodamine hydrochloride was injected intramuscularly to reduce peristaltic movement.

The initial sagittal and axial T2-weighted turbo spin echo (TSE) sequences were performed to determine the location of the rectal tumor. For APTw imaging, we used the Philips product implementation. More specifically, axial APTw images were acquired using a 3D TSE mDIXON sequence. The B1 field strength was 2 µT; continuous RF saturation pulse train had a duration of 2 s. The entire z-spectrum contains nine images acquired at various saturation frequency offsets, including ±3.5, ± 3.42, ± 3.58, and −1560 ppm. To enhance the signal-to-noise ratio in the APTw images, three of the Z-spectral images are acquired at +3.5 ppm using different echo shifts on the order of 0.5 ms. This allows us to calculate a B0 field map directly from APTw image acquisition *via* the mDIXON algorithm. Correction of B0 field homogeneity was achieved by a Lagrange interpolation among the different saturation frequency offsets on a voxel-by-voxel basis. mDIXON was applied to suppress lipid artifacts in APTw images. Other imaging parameters were as follows: repetition time (TR)/echo time (TE): 5864 ms/10 ms; field of view (FOV) 250 × 346; section thickness 5 mm; voxel size 1.8 × 1.8 × 5 mm; TSE factor 35.

Other MR sequences included high-resolution T2-weighted imaging (Turbo SE, TR/TE: 3900/100 ms, flip angle 90°, FOV 200 × 200 mm, section thickness 3 mm, matrix 288 × 228, TSE factor 17) in the axial, coronal, and sagittal planes; conventional axial DWI (echo planar SE, TR/TE 3000/72 ms, flip angle 90°, b = 0, 1000 s/mm^2^, section thickness 3 mm, matrix 82 × 82); plain and gadolinium-enhanced T1-weighted (turbo spin echo, TR/TE 578/10 ms, FOV 240 × 240 mm, section thickness 3 mm, matrix 300 × 230) in the axial, coronal, and sagittal planes.

### Image Analysis

All raw data were transferred to an Intellispace Portal workstation (Philips Healthcare, Best, the Netherlands). According to the principle of the APT algorithm, APTw signal was defined as the asymmetric magnetization transfer ratio (MTR_asym_) at 3.5ppm from the corrected Z spectrum and displayed as amide proton transfer weighted percentage.

$$MTR_{asym}(3.5ppm)=\frac{S_{sat}(-3.5ppm)-S_{sat}(+3.5ppm)}{S_{0}},$$

where MTR_asym_ [+3.5 ppm] is magnetization transfer ratio (MTR) asymmetry at +3.5 ppm offset frequency, and S_sat_ and S_0_ are the signal intensity acquired with and without selective saturation, respectively.

APTw SI=MTRasym [Δω = +3.5 ppm](%).

The apparent diffusion coefficient (ADC) was calculated from two DWI image sets of different b values (b = 0, 1000 s/mm^2^).

Image analysis was performed in consensus by two radiologists (YY and LM, with 20 and 10 years of experience in rectal cancer MRI, respectively) identified rectal lesions from T2WI together with DWI images. For quantitative analysis of APT SI, MRIcro software was used for manual segmentation of the rectal tumor. The outline of the rectal tumor was drawn manually with a freehand tool on high-resolution T2WI images and defined as the region of interest, avoiding the intestinal cavity. The region of interest was then copied to the corresponding APT image to obtain the average APT SI. The mean APT SI values of all slices were recorded for further analysis.

The changes in APT (ΔAPT) and ADC (ΔADC) values were defined as the difference between the corresponding post and pre-values.

### Tumor Volume Evaluation

Tumor volume was measured before and after NCRT by manually drawing the tumor margin with a PACS system (YLZ Ruitu Information Technology, Guangzhou, China) on T2-weighted images comprising the continuous tumor-containing image. The whole-tumor volume was then calculated by adding up each cross-sectional volume. Two radiologists (YY and LM) assessed the images in consensus. Tumor volume reduction (Δ tumor volume) was calculated as follows:

Δ tumor volume=pre-tumor volume−post-tumor volume

### Carcinoembryonic Antigen (CEA) Level Evaluation

Serum CEA levels were measured by the chemiluminescent method. The normal range of CEA is < 5ng/ml. Serum CEA levels before (pre-CEA) and after NCRT (post-CEA) were assessed approximately one week before CRT and within one week before surgery, respectively. The reduction of CEA was calculated as follows: ΔCEA = post-CEA – pre-CEA.

### Histological Analysis

All resected specimens were fixed in buffered formalin, embedded in paraffin and then made into 4-μm tissue sections for pathologic diagnosis. A pathological evaluation was performed by one pathologist (HS, with 21 years of experience). The pathologic tumor staging and tumor response to CRT were assessed according to the criteria described in the American Joint Committee on Cancer’s Cancer Staging Manual (AJCC 8^th^ edition) (30). The grade of tumor response to CRT was classified into four categories: TRG 0 (complete regression): no residual cancer cells; TRG 1 (near-complete regression): single or small groups of cancer cells; TRG 2 (moderate regression): residual cancer with desmoplastic response; TRG 3 (minimal regression): minimal evidence of tumor response. Patients with TRG 0–1 were considered to have a good response, whereas those with TRG 2–3 were considered to show a poor response to CRT (30).

### Statistical Analysis

Statistical analysis was performed using SPSS 20.0 (IBM, Armonk, New York) and MedCalc Statistical Software version 19.1.2 (MedCalc Software bv, Ostend, Belgium; https://www.medcalc.org; 2019).

The inter-class correlation coefficient was used to assess inter-observer agreement for the measurement of APT values, ADC values, and tumor volume values before and after NCRT. Inter-class correlation coefficient estimates above 0.75 were considered to have good reliability.

The Shapiro-Wilk test was used to determine the normality of data distribution. The paired t-test (normal distribution) and Wilcoxon signed-rank test (normality test failed) were used to assess the changes in APT, ADC, tumor volume, and CEA level between pre-NCRT and post-NCRT. A two-sample t-test and Mann-Whitney U test were used to assess the difference in the variances between good responders and poor responders. Logistic regression analysis was used to combine pre-APT and pre-ADC values to build a multi-parametric model. The Hosmer- Lemeshow test was used to measure the goodness- of- fit of the multivariate logistic model, and odds ratio (OR) and 95% CI was calculated.

Receiver operating characteristic (ROC) curve analysis was performed to evaluate the ability of six MR parameters (pre-APT& pre-ADC, pre-APT, pre-ADC, ΔAPT, ΔADC and Δ tumor volume) in discriminating good responders from poor responders. The sensitivity, specificity, positive predictive value (PPV), and negative predictive value (NPV) were calculated. A pairwise comparison of receiver-operating-characteristic curves was applied to test for significant differences between the areas under six receiver-operating-characteristic curves. A statistically significant difference was defined to be *p* < 0.05.

## Results

### Patient Characteristics

Eventually, 53 patients (average age, 60.2 years; range, 31–85 years) met the inclusion criteria and were enrolled in this study, consisting of 15 females and 38 males. The distribution of patients’ characteristics, including age, sex, histologic grade, TNM stage, and TRG status, is shown in **Table 1**. Of the 53 patients, 19 patients were defined as good responders (TRG 0–1) and 34 patients as poor responders (TRG 2-3).

**Table 1:** Clinic pathologic characteristics of enrolled patients.

Characteristic	Number of Patients
**Age (y)**	31-85y (60.2±12.6y)*
**Gender**	
Male	38
Female	15
**Clinical Stage before NCRT**	
cT3N+	26
cT4N0	16
cT4N+	11
**Histological grade**	
G1 (Well differentiated)	
G2 (Moderately differentiated)	45
G3 (Poorly differentiated)	8
**ypT Stage**	
T2	10
T3	36
T4	7
**ypN Stage**	
N0	16
N1a	20
N1b	11
N1c	N/A
N2a	6
N2b	N/A
**Tumor regression grade (TRG)**	
TRG 0	6
TRG 1	13
TRG 2	27
TRG 3	7
**Good Responders**	19
**Bad Responders**	34

Unless otherwise indicated, data are numbers of patients. *Data are mean ± standard deviation (range). Staging of tumors and TRG were in accordance with American Joint Committee on Cancer TNM classification. Grading of tumors was based on the WHO grading criteria.

### Inter-Observer Agreement

The interclass correlation coefficient of the two observers’ measurements were 0.934 (95% CI: 0.876–0.966) for pre-APT, 0.856 (95% CI: 0.739–0.923) for post-APT, 0.840 (95% CI:0.711–0.915) for pre-ADC, 0.862 (95% CI: 0.748–0.926) for post-ADC, 0.990 (95% CI: 0.981–0.995) for pre-volume, and 0.973 (95% CI 0.946–0.986) for post-volume. The two observers’ measurements of APT values, ADC values and tumor volumes showed strong agreement (**Table 2**).

**Table 2:** ICC for APT, ADC and volume values before and after NCRT measured by two radiologists.

	Pre-APT	Post-APT	Pre-ADC	Post-ADC	Pre-Volume	Post-Volume
**ICC (95% CI)**	0.934 (0.876-0.966)	0.856 (0.739–0.923)	0.840 (0.711-0.915)	0.862 (0.748-0.926)	0.990 (0.981-0.995)	0.973 (0.946-0.986)

ICC, interclass correlation coefficient; 95% CI, 95% confidence interval.

### Comparison of APT, ADC, Tumor Volume, and CEA Level Between Pre- and Post-NCRT

**Figure 2:**
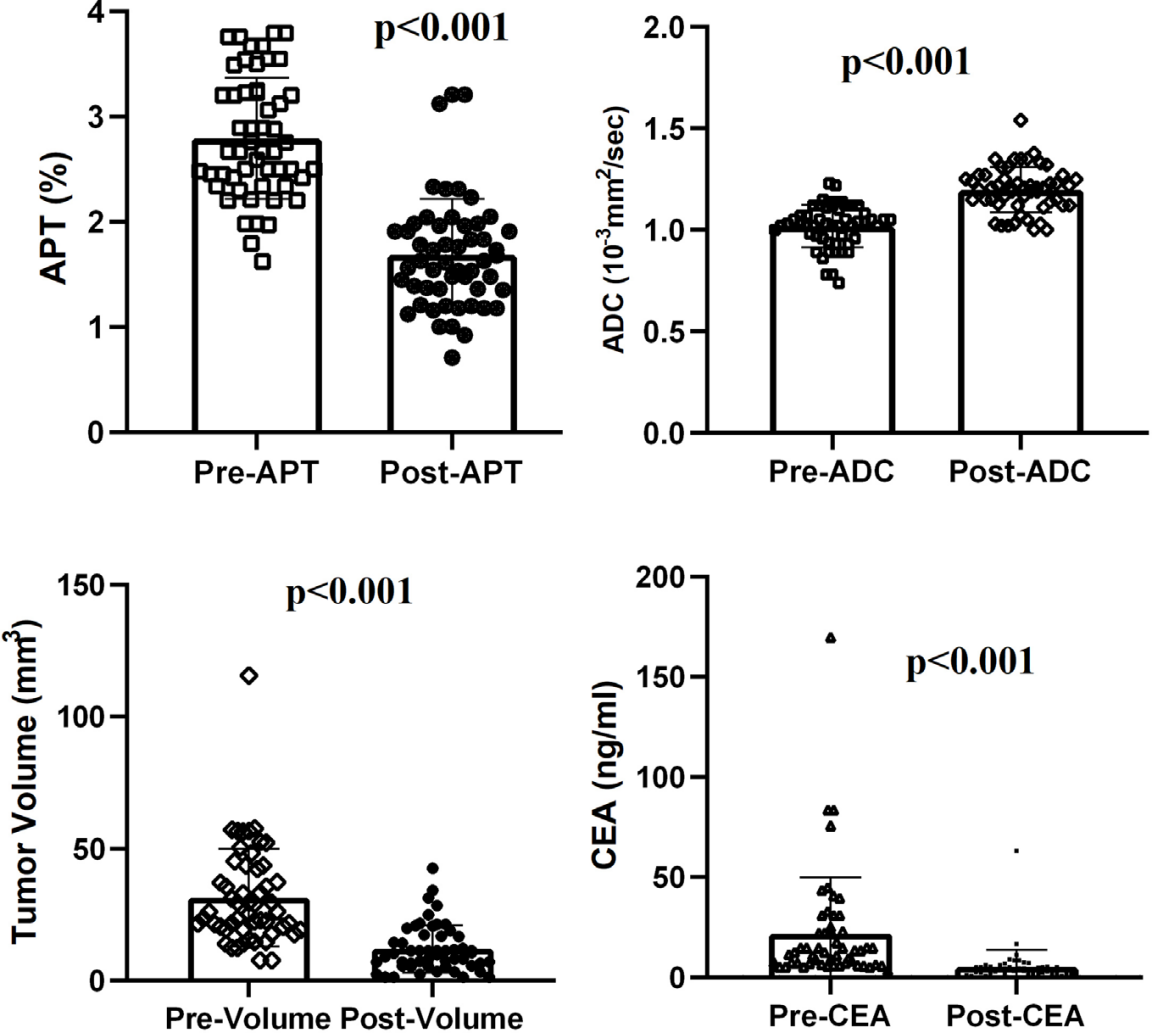
Comparison of APT, ADC, tumor volume, and CEA level between pre- and post-NCRT.

After NCRT, all the rectal tumors had lower APT values (2.794 ± 0.575 vs 1.687 ± 0.527, t = 12. 315, p < 0.001) and higher ADC values (1.020 ± 0.105 10^-3^mm^2^/s vs 1.120 ± 0.111 10^-3^mm^2^/s, t = -10.475, p < 0.001). The tumor volume decreased significantly from a median of 31.95 cm^3^ (range 7.68–115.60 cm^3^) before NCRT to a median of 11.73 cm^3^ (range 1.25–42.50 cm^3^) after NCRT (z = -6.334, p < 0.001). A median volume reduction rate of 63.3% was found. The CEA level decreased significantly from a median of 21.87ng/ml (range of 3.26–169.70 ng/ml) before NCRT to a median of 5.30 ng/ml (range 0.81–63.04 ng/ml) after NCRT (z = -6.335, p < 0.001) (**Figure 2**).

### Parameter Comparison Between Good and Poor Responders to NCRT

**Figure 3:**
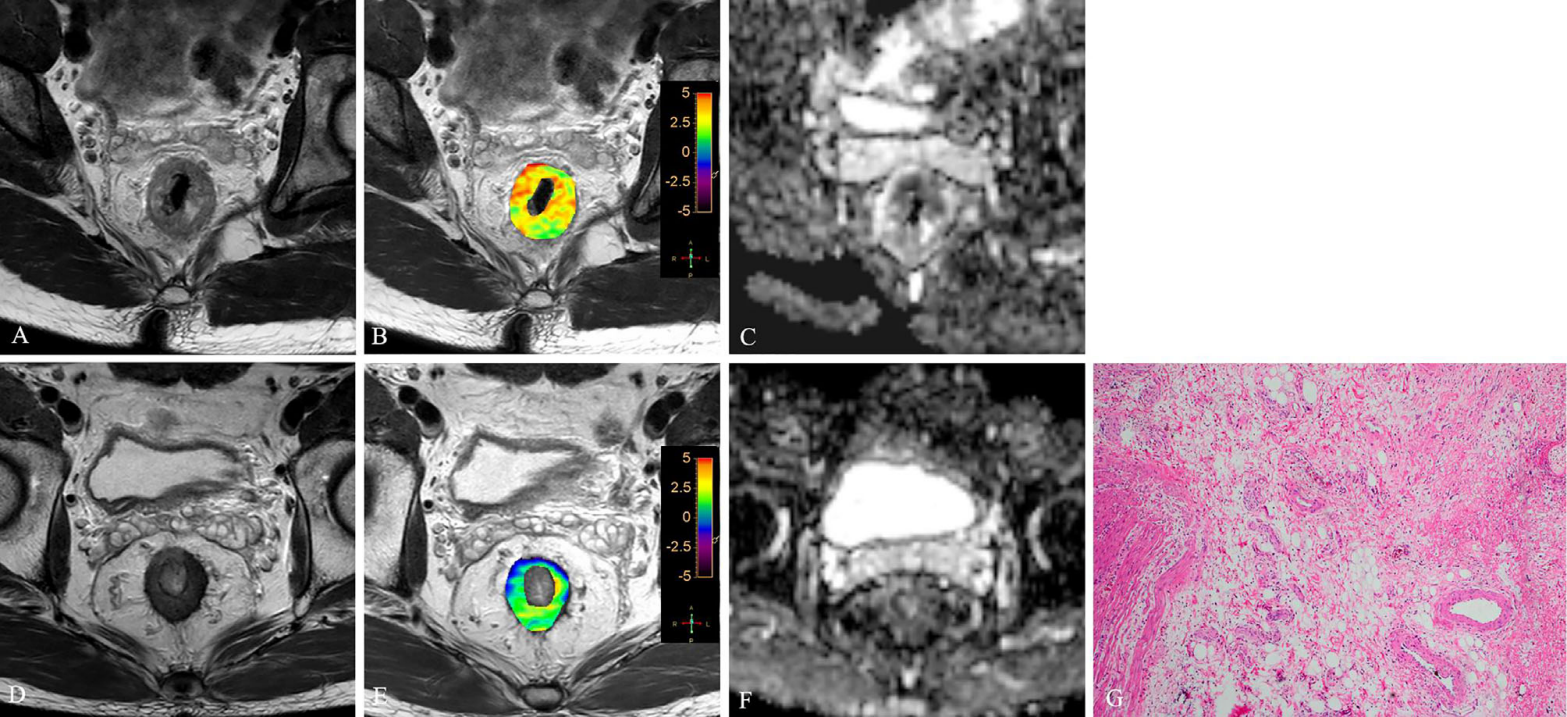
T2WI **(A, D)**, APTw **(B, E)** and ADC map **(C, F)** of a 63-year-old male with LARC before and after NCRT. At 8 weeks after NCRT, compared to the MR performed at baseline **(A–C)**, the mean APTw SI decreased from 3.23% **(B)** to 1.54%, the mean ADC value increased from 1.042×10^-3^mm^2^/s **(C)** to 1.112×10^-3^mm^2^/s **(F)**. Histopathological examination after surgery shows the degree of tumor regression is TRG 0 (H. E staining, ×40, **G**).

**Figure 4:**
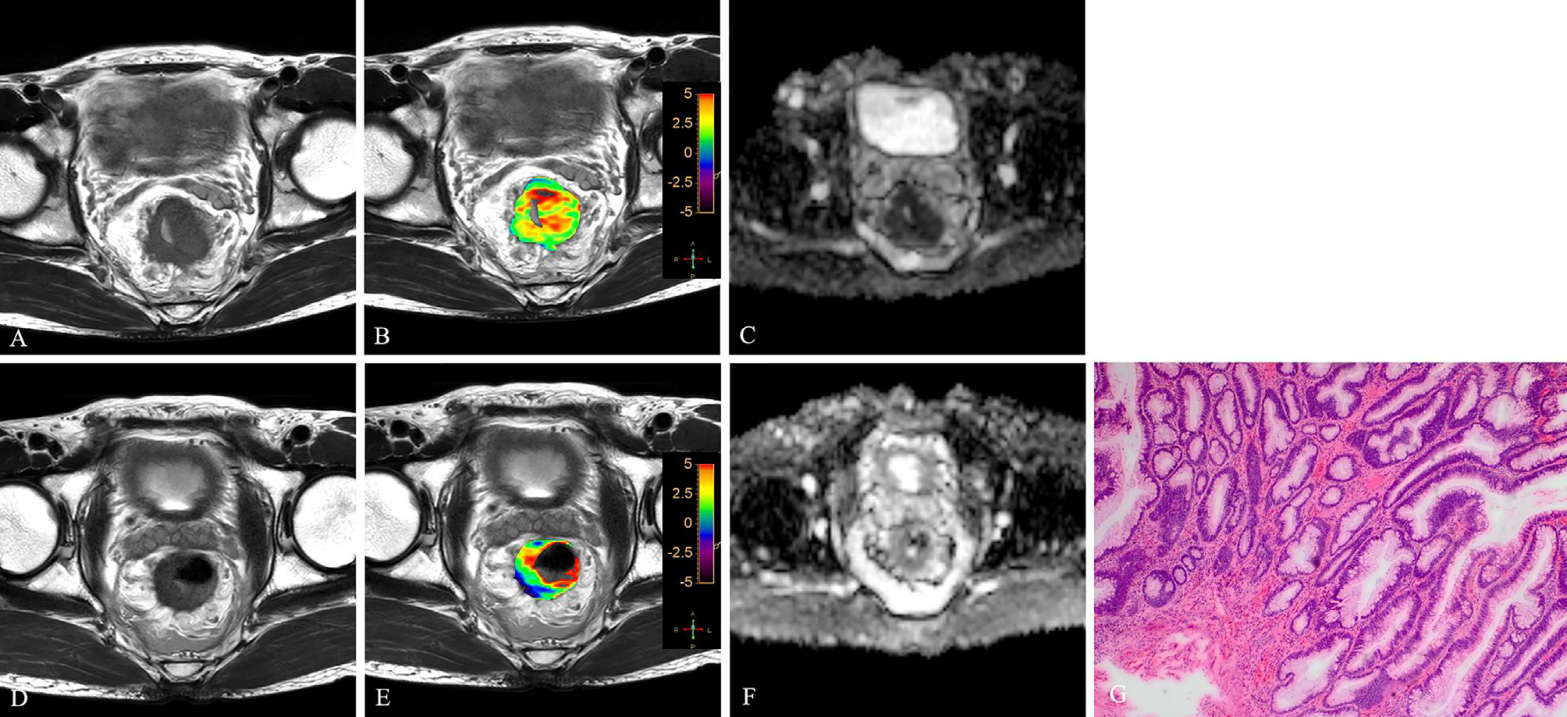
T2WI **(A, D)**, APTw **(B, E)** and ADC map **(C, F)** of a 56-year-old male with LARC before and after NCRT. At 8 weeks after NCRT, compared to the MR performed at baseline **(A–C)**, the mean APTw SI decreased from 3.20% **(B)** to 1.614%, the mean ADC value increased from 0.89×10^-3^mm^2^/s **(C)** to 1.234×10^-3^mm^2^/s **(F)**. Histopathological examination after surgery shows the degree of tumor regression is TRG 1 (H. E staining, ×40, **G**).

**Figure 5:**
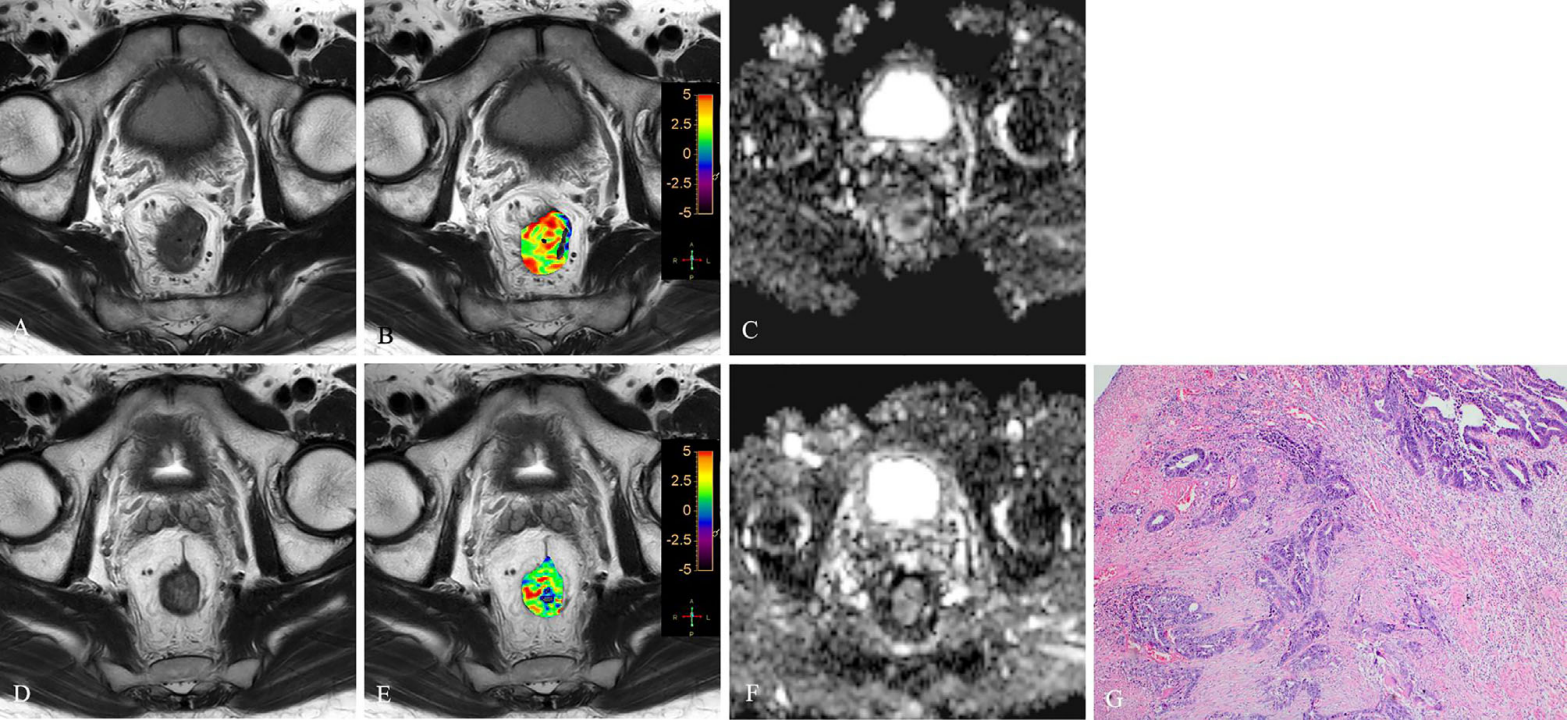
T2WI **(A, D)**, APTw **(B, E)** and ADC map **(C, F)** of a 51-year-old female with LARC before and after NCRT. At 6 weeks after NCRT, compared to the MR performed at baseline **(A–C)**, the mean APTw SI decreased from 2.20% **(B)** to 1.783%, the mean ADC value increased from 1.120×10^-3^mm^2^/s **(C)** to 1.205×10^-3^mm^2^/s **(F)**. Histopathological examination after surgery shows the degree of tumor regression is TRG 3 (H. E staining, ×40, **G**).

**Figure 6:**
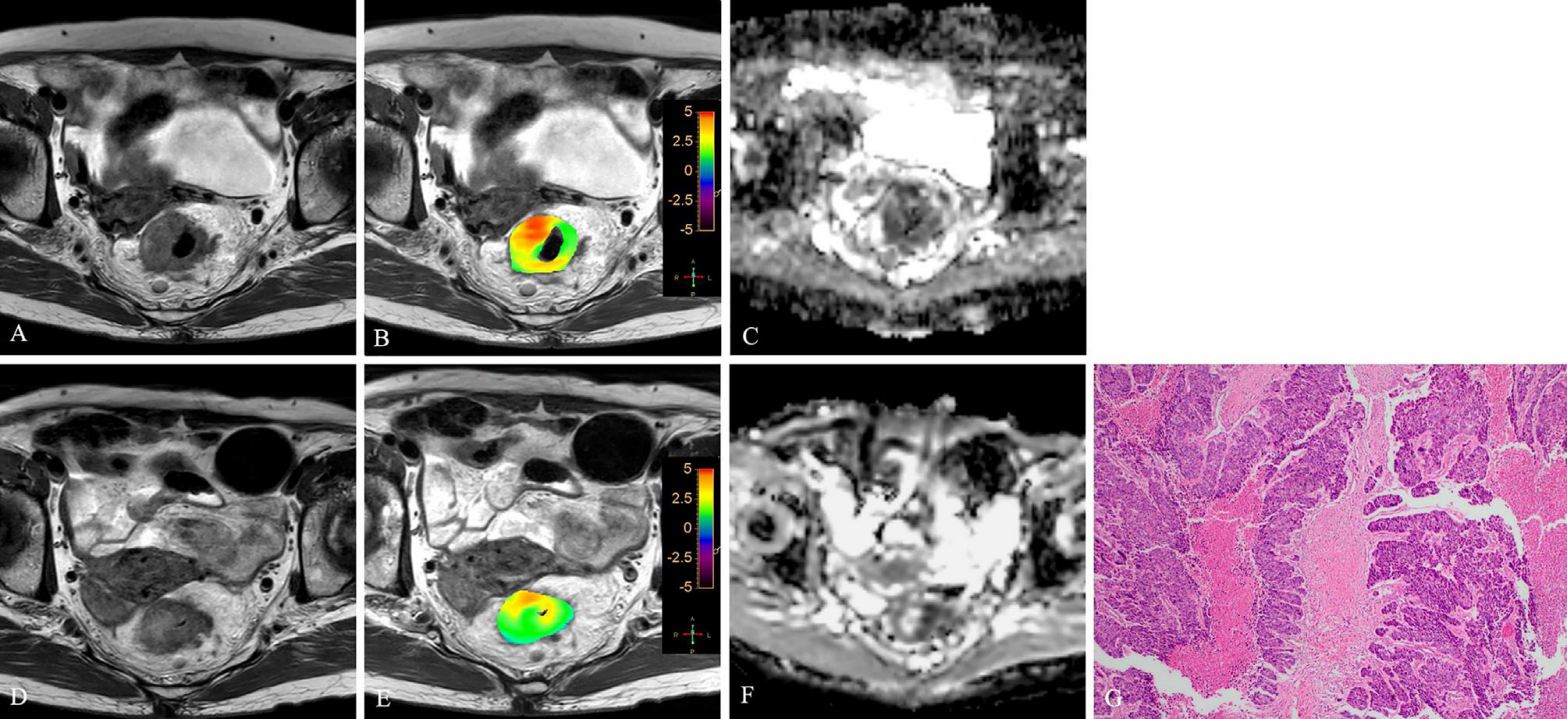
T2WI **(A, D)**, APTw **(B, E)** and ADC map **(C, F)** of a 47-year-old male with LARC before and after NCRT. At 6 weeks after NCRT, compared to the MR performed at baseline **(A–C)**, the mean APTw SI decreased from 2.50% **(B)** to 1.83%, the mean ADC value increased from 1.040×10^-3^mm^2^/s **(C)** to 1.245×10^-3^mm^2^/s **(F)**. Histopathological examination after surgery shows the degree of tumor regression is TRG 3 (H. E staining, ×40, **G**).

Significant differences were found between good and poor responders for pre-APT values, Δ APT, pre-ADC, and Δ tumor volume. The good responder group demonstrated higher pre-APT values, higher ΔAPT values, lower pre-ADC values and higher Δ tumor volumes than the poor responder group (**Figures 3**–**6**). However, no difference was found in post-APT values, post-ADC values, ΔADC values, pre-volumes, post-volumes, pre-CEA levels, post-CEA levels and ΔCEA levels between the good and poor responder groups (all p > 0.05) (**Table 3**).

**Table 3:** Comparison of variables between good and poor responders.

	Responders	Two-sample T test (Mean± SD)	Mann-Whitney U Test (Mean Rank)	Z	*P value*
**Pre-APT (%)**	Good (n=19)	3.222 ± 0.516			<0.001
	Poor (n=34)	2.554 ± 0.459			
**Post-APT (%)**	Good (n=19)		28.26	-0.045	0.656
	Poor (n=34)		32.00		
**Δ APT (%)**	Good (n=19)		18.05	-3.154	0.002
	Poor (n=34)		32.00		
**Pre-ADC**	Good (n=19)	0.970 ± 0.107			0.009
**(10^-3^mm^2^/s)**	Poor (n=34)	1.047 ± 0.095			
**Post-ADC**	Good (n=19)	1.162 ± 0.118			0.09
**(10^-3^mm^2^/s)**	Poor (n=34)	1.216 ± 0.103			
**Δ ADC**	Good (n=19)		28.71	-0.604	0.546
**(10^-3^mm^2^/s)**	Poor (n=34)		26.04		
**Pre-Volume (mm^3^)**	Good (n=19)		30.63	-1.286	0.202
	Poor (n=34)		24.97		
**Post-Volume (mm^3^)**	Good (n=19)		25.97	-0.362	0.718
	Poor (n=34)		27.57		
**Δ Volume (mm^3^)**	Good (n=19)		33.11	-2.152	0.031
	Poor (n=34)		23.59		
**Pre-CEA (ng/ml)**	Good (n=19)		24.74	-0.795	0.425
	Poor (n=34)		28.26		
**Post-CEA (ng/ml)**	Good (n=19)		28.53	-0.538	0.591
	Poor (n=34)		26.15		
**Δ CEA (ng/ml)**	Good (n=19)		23.37	-1.280	0.201
	Poor (n=34)		29.03		

Data are means ± standard deviations (normal distribution), mean rank (normal distribution failed). ADC values are given in 10^-3^mm^2^/sec, volume is given in mm^3^, CEA level is given in ng/ml.

### Diagnostic Capacity of APT and ADC in Predicting NCRT Treatment Response

The significant level of the Hosmer-Lemeshow test was 0.245, ORs of pre-APT and pre-ADC were 0.044 (p<0.001), 0.001 (p=0.006), respectively, suggesting the fit of the model had good goodness.

**Figure 7:**
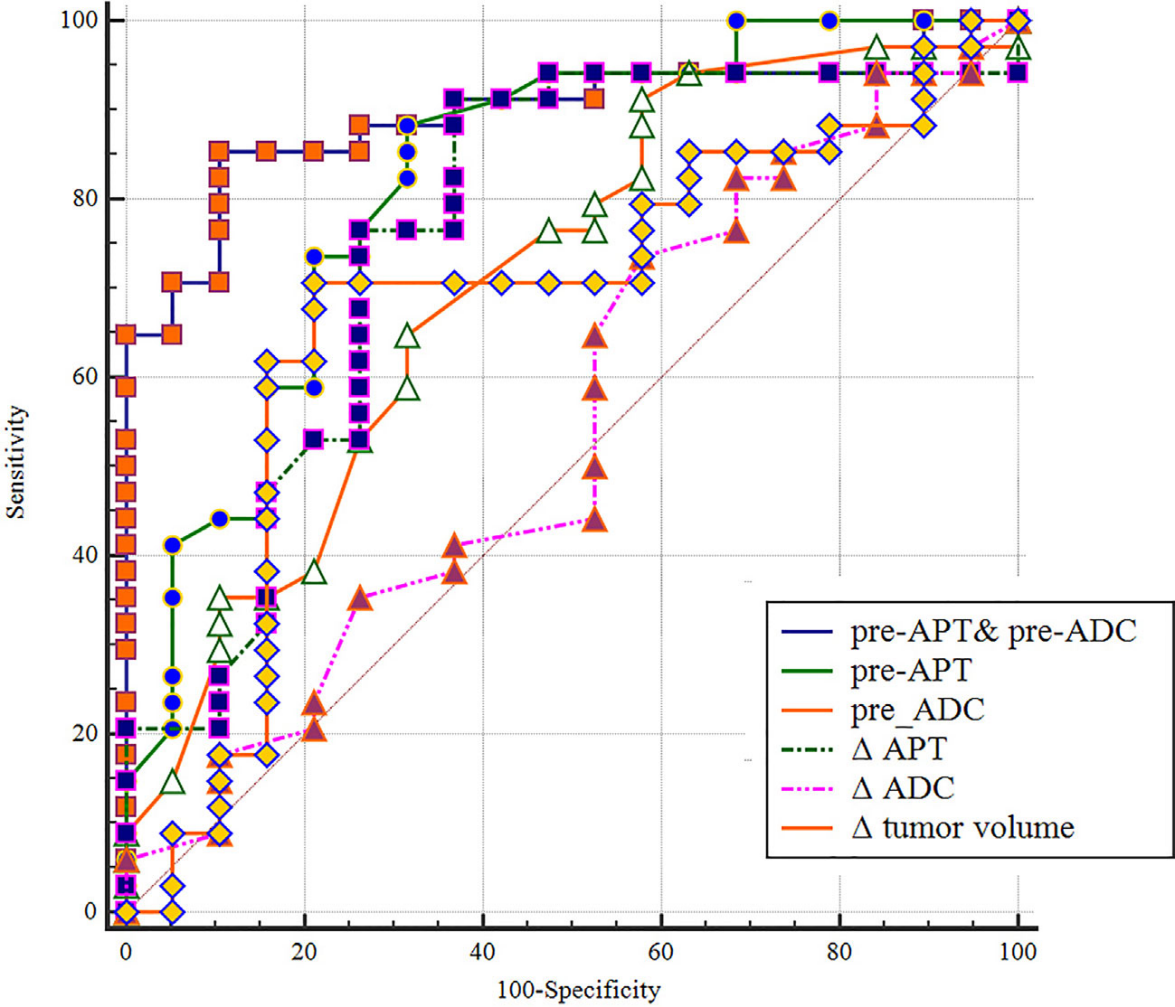
A comparison of the diagnostic capability in predicting in discriminating good responders from poor responders between pre-APT & pre-ADC, pre-APT, pre-ADC, ΔAPT, ΔADC and Δ tumor volume. ROC analysis shows pre-APT combine with pre-ADC has a higher AUC (0.895) than the other parameters.

The sensitivity, specificity, PPV, and NPV of pre-APT combined with pre-ADC, pre-APT, ΔAPT, pre-ADC value, ΔADC and Δ tumor volume for predicting the response to NCRT are summarized in **Table 4**. The AUCs were 0.895, 0.800, 0.778, 0.691, 0.543 and 0.680 for pre-APT& pre-ADC, pre-APT, pre-ADC, ΔAPT, ΔADC and Δ tumor volume, respectively. A combination of APT and ADC values before NCRT achieved a sensitivity of 85.29% and specificity of 89.47% for predicting a good response to NCRT, whereas PPV and NPV were 93.50% and 77.30%, respectively (**Table 4**). Compare to pre-ADC, ΔADC and Δ tumor volume, pre-APT combined with pre-ADC showed greater diagnostic performance (p=0.019, p<0.001and p= 0.013, respectively). However, there was no statistical difference among pre-APT & ADC, pre-APT, and Δ APT (all p>0.05) (**Figure 7**).

**Table 4:** Performance of different MR parameters in predicting NCRT response in the patients with LARC.

Parameter	AUC	*P* value	Youden Index	Sensitivity (%)	Specificity (%)	PPV (%)	NPV (%)
Pre-APT & Pre-ADC	0.895 (0.780-0.962)	<0.001	0.748	85.29	89.47	93.5	77.3
Pre-APT (%)	0.824 (0.694-0.915)	<0.001	0.517	88.24	68.42	88.3	76.5
Δ APT (%)	0.763 (0.626-0.869)	<0.001	0.543	91.18	63.16	81.6	80.0
Pre-ADC (10^-3^mm^2^/s)	0.707 (0.566-0.824)	0.007	0.333	91.18	42.11	73.8	72.7
Δ ADC (10^-3^mm^2^/s)	0.550 (0.408-0.687)	0.558	0.156	73.52	42.11	69.4	47.1
Δ tumor volume (mm^3^)	0.680 (0.537-0.801)	0.030	0.495	70.59	78.95	68.6	44.4

Data in parentheses are numerators and denominators and data in brackets are 95% confidence intervals. APT, amide proton transfer; ADC, apparent diffusion coefficient; AUC, area under curve; NPV, negative predictive value; PPV, positive predictive value.

## Discussion

In this study, we investigated the ability of APTw MRI combined with DWI to evaluate pathologic tumor down-staging and predict treatment responses after NCRT in rectal adenocarcinoma. After NCRT, all rectal tumors demonstrated significantly lower APT values and higher ADC values, as well as significantly smaller tumor volumes and lower CEA levels. APTw imaging is a new MRI contrast method based on CEST, using the signal of amide protons (NH groups) contained in proteins and peptides. It has been suggested that endogenous mobile cytoplasmic proteins are the major source of APT signals (31). NCRT causes a series of pathological changes in rectal cancer, including cellular damage, tumor necrosis, local inflammatory reaction, and fibrosis replacing tumor glands (32), which leads to a lower content of proteins and peptides than in viable tumors and thus is expected to demonstrate a lower APTw signal. However, the increase in ADC after NCRT is due to increased diffusion of water molecules caused by cell damage, tumor necrosis, and extracellular edema.

When we compared these parameters between different therapeutic effect groups, we noted that the good responders demonstrated significantly higher APT values and lower ADC values before NCRT than the poor responders. Significantly higher ΔAPT values were also observed in patients with good responses. We suggest that the following factors might have contributed to this phenomenon. First, a high APT value for the tumor was associated with cellular proliferation and proved to be positively correlated with Ki67 expression level (33, 34), which is a cellular marker for cell proliferation and growth. Cellular proliferation is relevant to radiation response. Rapidly proliferating cells are more susceptible to NCRT-induced damage than quiescent cells because they have less time to repair the damage. Studies found that patients with higher Ki67 expression are associated with better response and downstaging in highly proliferative tumors, exhibiting a greater tumor regression grade response and pCR rate (35, 36). Second, tumor proliferation also depends on angiogenesis. Better-perfused tumors demonstrated higher proliferation capability, and blood could also generate sufficient CEST contrast. Studies have found high blood flow and permeability in good responders. Increased blood flow and enhanced microvascular permeability in the tumor bed provide not only better access for chemotherapy, but also less hypoxia-mediated radio-resistance, which contribute to good responses to NCRT (37). DCE-MRI of rectal cancer demonstrated that tumors with better perfusion showed greater nodal and tumor downstaging after radiotherapy than poorly perfused tumors (38). However, our result on APT changes after NCRT contrasted with that of Nishie et al., who found that pretherapeutic mean APTw SI of low-response group was significantly higher than that of the high-response group (29). This difference may be explained by the fact that Nieshi et al. used a different TRG grading criteria, which is according to necrosis or cytological changes of the tumor. In contrast, the TRG system in our study is based on the evaluation of residual tumor cells. Additionally, single-slice APT MR sequence in Nieshi et al.’s study may not reflect complete characteristics of the tumor.

DWI has been extensively used for prediction of response to neoadjuvant treatment in rectal cancer, and has been recommended in international clinical guidelines as a valuable adjunct to a restaging MRI protocol. All studies reported that tumor ADC values increased after NCRT, which is due to radiation-induced cellular damage and necrosis (39). However, results regarding the use of ADC in predicting the NCRT response have been inconsistent. Some studies demonstrated significantly lower pre-NCRT ADC values in the good responders (40–42), whereas Monguzzi L et al. found no benefit of pre- treatment ADC in assessing NCRT response (13). These conflicting results may be attributed to variations in DWI protocol, end-points, and variabilities in the patient selection criteria. Besides, it has been difficult to identify treatment response accurately based on DWI alone (39).

Additionally, tumor size and CEA level have proven to be independent predictors of pCR to NCRT in rectal cancer. Based on the results of systemic review and meta-analysis, small tumor size and low levels of pre-treatment CEA are associated with pCR or good response (43, 44). We found a significant reduction of tumor volume in good responders, which is in agreement with previous studies (7, 8). Although in the present study the good responders have lower CEA levels before NCRT, the difference is not significant.

Accurate early prediction of the response to NCRT would aid in the stratification of patients into optimal therapy managements and improve therapeutic outcomes in rectal cancer. Concerning discriminating tumor response to NCRT, we demonstrated that pre-APT combined with pre-ADC had the highest AUC (0.895) among all parameters. This combined parameter, with 85.29% sensitivity and 89.47% specificity, achieved greater efficacy than pre-ADC (p=0.029), ΔADC (p<0.001), pre-APT and ΔAPT, although differences of AUCs between pre-APT& pre-ADC, pre-APT, and ΔAPT were not statistically significant.

Our study has several limitations. The primary limitations were small sample size and lack of external validation steps. Second, we used the American Joint Committee on Cancer system for evaluating tumor regression. However, several grading systems are proposed for tumor regression, which may yield variable results owing to their different TRG components and grading criteria. Third, our study did not include evaluation of lymph node involvement, and it is known that presence of lymph node metastasis can be found in patients with pCR. Fourth, different chemotherapy regimens in our study may have an impact on the therapy efficacy. Lastly, our interpretation of APTw signal changes and tumor regression was based on the quantity of residual tumor cells and cytoplasmic protein. However, pathologic changes after NCRT are diverse and complex. Besides reduced tumor cellularity, variable histology changes after NCRT—including submucosal fibrosis, mucin pool formation, and calcification—could affect APT signal changes. Therefore, further research should be conducted to explain the biophysical sources of altered APT changes in rectal cancer after NCRT.

In conclusion, the combination of APTw MRI and DWI before NCRT holds potential in evaluating NCRT response of LARC, as is the capability to detect changes in cellular protein and cellularity density noninvasively, might provide additional information for clinical decision making in the management after NCRT.

## Data Availability Statement

The original contributions presented in the study are included in the article/**Supplementary Material**. Further inquiries can be directed to the corresponding author.

## Ethics Statement

The studies involving human participants were reviewed and approved by The Second Affiliated Hospital, Guangzhou University of Chinese Medicine. The patients/participants provided their written informed consent to participate in this study.

## Author Contributions

The authors confirm contribution to the paper as follows: study conception and design: XL, WC, and LL. Data collection: QW, JP, and SH. Analysis and interpretation of results: WC, LM, and YY. Draft manuscript preparation: WC, BL, and XL. All authors contributed to the article and approved the submitted version.

## Supplementary Material

The Supplementary Material for this article can be found online at: https://www.frontiersin.org/articles/10.3389/fonc.2021.698427/full#supplementary-material

## Conflict of Interest

The authors declare that the research was conducted in the absence of any commercial or financial relationships that could be construed as a potential conflict of interest.

## Abbreviations

ADC, apparent diffusion coefficient; APT, amide proton transfer; AUC, area under the curve; CEST, chemical exchange saturation transfer; DCE, dynamic contrast enhanced; DWI, diffusion weighted; LARC, locally advanced rectal cancer; NCRT, neoadjuvant chemoradiotherapy; ROC, receiver operating characteristic; ROI, region of interest; SD, standard deviation; pCR, pathologic complete response; TRG, tumor regression grade.
