# Supplementary material for: Predicting Treatment Response of Neoadjuvant Chemoradiotherapy in Locally Advanced Rectal Cancer Using Amide Proton Transfer MRI Combined With Diffusion-Weighted Imaging
Source: Front Oncol. 2021 Jul 1;11:698427. doi: 10.3389/fonc.2021.698427 (PMC8281887; doi:10.3389/fonc.2021.698427)
Supplement: Supplementary file 1 [file Table_1.docx]

**Comparison of ROC curves**

| **Variable 1** | Pre-APT& Pre-ADC |
| --- | --- |
| **Variable 2** | Pre-APT |
| **Variable 3** | Pre-ADC |
| **Variable 4** | **Δ** APT |
| **Variable 5** | **Δ** ADC |
| **Variable 6** | **Δ** Volume |
| **Classification** | Response |

| **Sample size** | 53 |
| --- | --- |
| **Positive group a** | 34 (64.15%) |
| **Negative group b** | 19 (35.85%) |

a Response = 1

b Response = 0

| **Variable** | **AUC** | **SEa** | **95% CI** |
| --- | --- | --- | --- |
| **Pre- APT &Pre-ADC** | 0.895 | 0.0446 | 0.780 to 0.962 |
| **Pre-APT** | 0.824 | 0.0631 | 0.694 to 0.915 |
| **Pre-ADC** | 0.707 | 0.0768 | 0.566 to 0.824 |
| **ΔAPT** | 0.763 | 0.0740 | 0.626 to 0.869 |
| **ΔADC** | 0.550 | 0.0860 | 0.408 to 0.687 |
| **Δ Volume** | 0.680 | 0.0819 | 0.537 to 0.801 |

 a DeLong et al., 1988

 b Binomial exact

**Pairwise comparison of ROC curves**

| **Pre- APT& Pre-ADC Vs Pre-APT** | |
| --- | --- |
| Difference between areas | 0.0712 |
| Standard Error a | 0.0480 |
| 95% Confidence Interval | -0.0230 to 0.165 |
| z statistic | 1.482 |
| Significance level | P = 0.1383 |
| **Pre-APT& Pre-ADC Vs Pre-ADC** | |
| Difference between areas | 0.188 |
| Standard Error a | 0.0794 |
| 95% Confidence Interval | 0.0325 to 0.344 |
| z statistic | 2.369 |
| Significance level | P = 0.0178 |
| **Pre-APT& Pre-ADC Vs ΔAPT** |  |
| Difference between areas | 0.132 |
| Standard Error a | 0.0641 |
| 95% Confidence Interval | 0.00597 to 0.257 |
| z statistic | 2.053 |
| Significance level | P = 0.0401 |
| **Pre-APT& Pre-ADC Vs Δ ADC** | |
| Difference between areas | 0.344 |
| Standard Error a | 0.0902 |
| 95% Confidence Interval | 0.168 to 0.521 |
| z statistic | 3.818 |
| Significance level | P = 0.0001 |
| **Pre- APT& Pre-ADC Vs Δ Volume** | |
| Difference between areas | 0.215 |
| Standard Error a | 0.0862 |
| 95% Confidence Interval | 0.0462 to 0.384 |
| z statistic | 2.496 |
| Significance level | P = 0.0126 |
| **Pre- APT Vs pre- ADC** | |
| Difference between areas | 0.117 |
| Standard Error a | 0.114 |
| 95% Confidence Interval | -0.106 to 0.340 |
| z statistic | 1.027 |
| Significance level | P = 0.3042 |
| **Pre-APT Vs Δ APT** | |
| Difference between areas | 0.0604 |
| Standard Error a | 0.0740 |
| 95% Confidence Interval | -0.0846 to 0.205 |
| z statistic | 0.816 |
| Significance level | P = 0.4145 |
| **Pre-APT Vs Δ ADC** |  |
| Difference between areas | 0.273 |
| Standard Error a | 0.108 |
| 95% Confidence Interval | 0.0606 to 0.486 |
| z statistic | 2.519 |
| Significance level | P = 0.0118 |
| **Pre-APT Vs Δ Volume** |  |
| Difference between areas | 0.144 |
| Standard Error a | 0.0806 |
| 95% Confidence Interval | -0.0140 to 0.302 |
| z statistic | 1.787 |
| Significance level | P = 0.0740 |
| **Pre-ADC Vs ΔAPT** |  |
| Difference between areas | 0.0565 |
| Standard Error a | 0.103 |
| 95% Confidence Interval | -0.145 to 0.258 |
| z statistic | 0.549 |
| Significance level | P = 0.5828 |
| **Pre-ADC Vs ΔADC** |  |
| Difference between areas | 0.156 |
| Standard Error a | 0.0954 |
| 95% Confidence Interval | -0.0306 to 0.343 |
| z statistic | 1.639 |
| Significance level | P = 0.1012 |
| **Pre-ADC Vs Δ Volume** | |
| Difference between areas | 0.0271 |
| Standard Error a | 0.121 |
| 95% Confidence Interval | -0.209 to 0.264 |
| z statistic | 0.224 |
| Significance level | P = 0.8224 |
| **Δ**APT vs **Δ** ADC | |
| Difference between areas | 0.213 |
| Standard Error a | 0.114 |
| 95% Confidence Interval | -0.00980 to 0.435 |
| z statistic | 1.874 |
| Significance level | P = 0.0610 |
| **ΔAPT Vs Δ Volume** | |
| Difference between areas | 0.0836 |
| Standard Error a | 0.110 |
| 95% Confidence Interval | -0.133 to 0.300 |
| z statistic | 0.757 |
| Significance level | P = 0.4492 |
| **Δ ADC Vs Δ Volume** |  |
| Difference between areas | 0.129 |
| Standard Error a | 0.117 |
| 95% Confidence Interval | -0.0998 to 0.358 |
| z statistic | 1.106 |
| Significance level | P = 0.2687 |

**a DeLong et al., 1988**
